# Risk Factors Associated with Surgical Site Infection following Cesarean Section in Tertiary Care Hospital, Nepal

**DOI:** 10.1155/2022/4442453

**Published:** 2022-05-16

**Authors:** Astha Regmi, Neebha Ojha, Meeta Singh, Asmita Ghimire, Nisha Kharel

**Affiliations:** ^1^Department of Obstetrics and Gynecology, Damauli Hospital, Tanahun, Nepal; ^2^Department of Obstetrics and Gynecology, TUTH, IOM, Nepal; ^3^Department of Obstetrics and Gynecology, NGMCTH, Nepal; ^4^Department of Obstetrics and Gynecology, Grande International Hospital, Kathmandu, Nepal

## Abstract

**Background:**

Cesarean section (CS) is one of the most performed surgeries in obstetrics. Surgical site infection is the major cause of morbidity and mortality causing an increase in the duration of hospitalization as well as the cost of admission for the patient.

**Objective:**

To determine incidence of surgical site infection following cesarean section, classify them according to CDC criteria, and identify the different risk factors. *Methodology*. This is a case-control study conducted at the Department of Obstetrics and Gynecology at Tribhuvan University Teaching Hospital (TUTH), main campus of Institute of Medicine (IOM), Kathmandu, Nepal. Surgical site infections (SSI) in patients who underwent cesarean sections from February 2019 to August 2019 were taken as cases, while the patients who underwent cesarean section before or after the procedure and did not develop SSI comprised the controls. Visual inspection during ward rounds, reports from laboratory, and postprocedure follow-ups for up to 30 days formed the basis of identifying infections on the patients. Risk factors were identified by bivariate and multivariate logistic regression.

**Results:**

Out of 1135 cases of cesarean sections, 97 of them developed SSI with incidence rate of 8.54%. Among them, 94.85% were superficial incisional and 5.15% were deep incisional type of SSI with no organ space type. Cases had higher mean age 26.88 ± 4.38 years compared to 24.81 ± 5.08 years in controls. Host-related risk factors which led to higher odds of developing surgical site infection (SSI) were obesity with adjusted odds ratio (AOR) 15.72 (confidence interval (CI): 4.60-53.67), diabetes/hypertension in pregnancy with AOR 4.75(CI 1.69-13.32), and other medical diseases with AOR 9.38 (CI 2.89-30.46). Duration of the rupture of membrane for more than 18 hours with AOR 8.38 (CI 1.48-47.35), more than five per vaginal (PV) examination with AOR 1.93 (95% CI 1.03-3.64), and in labor status with AOR 6.52 (CI 1.17-36.38) were some procedure-related factors resulting into higher odds of infection.

**Conclusion:**

Multiple risk factors like age, obesity, medical complications during pregnancy, occurrence of labor status during cesarean section, prolonged duration of rupture of membrane for more than 18 hours, and more than five vaginal examinations before the procedure increases the chance of surgical site infection (SSI) following cesarean section.

## 1. Introduction

Cesarean section (CS) is one of the most performed major surgical procedures in obstetrics. When adequately indicated, it can prevent poor obstetric outcomes and can be a life-saving procedure for both the mother and the fetus. Nevertheless, at a time when the cesarean delivery rate has been rising globally, concern is growing about the risk of maternal mortality and morbidity that comes with it parallelly. Major complications associated with cesarean delivery include postpartum endometritis, hemorrhage, injury to pelvic organs, thromboembolic disorders, anesthesia-related complications, and wound complications [1, 2].

Surgical site infection (SSI) is an infection that occurs within 30 days after the operation and involves the skin and subcutaneous tissue of the incision (superficial incisional) and/or the deep soft tissue (for example, fascia and muscle) of the incision (deep incisional) and/or any part of the anatomy (for example, organs and spaces) other than the incision that was opened or manipulated during an operation (organ/space) [3]. It is the most common infection in surgical patients and a major cause of maternal morbidity and mortality too [4].

The recent incidence rates of SSI following cesarean section in different countries are India 13% [1], Nepal 12.6% [5], Nigeria 9.1% [6], Tanzania 10.9% [7], and Australia 17% [8]. In a systematic review of the maternal intrinsic risk factors associated with SSI following cesarean section, obesity and chorioamnionitis were concluded to be common risk factors for the overall SSI [8]. Other factors are duration of rupture of membranes, emergency CS [9], lack or improper use of preoperative prophylaxis antibiotics [9–11], and onset of labor [12]. Cesarean section in the Tribhuvan University Teaching Hospital (TUTH) has escalated from 30.3% to 46.7% in the last ten years. As a result, higher numbers of SSIs can be expected. Readmission of postpartum women in the hospital not only adds burden to the hospital and healthcare staff but also causes the huge impact on psychosocial health of a mother. Therefore, identifying risk factors for SSI in a hospital setting might be of importance to reduce maternal morbidity and mortality. However, there are very few studies about SSI following CS in Nepal. This had led the healthcare system to be unaware of many risk factors which might be more prevalent in our setting.

The main objective of this study is to determine the incidence of SSI following CS in TUTH, classify them, and analyze different host, pregnancy, and procedure-related risk factors.

## 2. Methodology

### 2.1. Study Setting and Design

This is a case-control study conducted in the Department of Obstetrics and Gynecology, Institute of Medicine, Tribhuvan University Teaching Hospital, Kathmandu, for duration of 7 months from February 2019 to October 2019. TUTH is in capital city of Nepal and is one the tertiary care centers for obstetric patients' referral causing a huge patient flow.

### 2.2. Sample Size Determination and Sampling Technique

The sample size was calculated using STATA 14.1 based on previous study by Dagshinjav et al. (2016) in Mongolia [13]. Based on their methodology, the sample size in this study was calculated. The sample size was determined in the following manner.

Two-sided confidence level = 95%

Power (1 − *β*) = 90%

Ratio of control to cases = 1 : 1

Percent of controls exposed = 52.6%

Expected odds ratio: 2.7

According to Dagshinjya et al. (2016), odds ratio of postcesarean surgical site infection was 2.7 and percent of control exposed was 52.6%

Sample size for case = 72

Sample size for control = 72

Thus, total sample size = 144 with 10% nonresponse rate, and the total sample size would be 160 (80 cases and 80 control)

### 2.3. Operational Definitions

(I) Surgical site infection (SSI): the Centers for Disease Control and Prevention (CDC) defines surgical site infection (SSI) as an infection following surgery at the part of the body where the surgery was conducted
(II) Superficial SSI: infection occurs within 30 days after the operation and only involves skin and subcutaneous tissue at the region of incision and at least one of the following conditions occurs:
  - Purulent drainage with or without laboratory confirmation, from the superficial incision
  - Organisms isolated from an aseptically obtained culture of fluid or tissue from the superficial incision
  - At least one of the following signs or symptoms of infection: pain or tenderness, localized swelling, redness, or heat and superficial incision is deliberately opened by surgeon, unless incision is culture negative
  - Diagnosis of superficial incisional SSI made by a surgeon or attending physician
(III) Deep incisional SSI: infection occurs within 30 days after the operation if no implant is left in place or within one year if implant is in place and the infection appears to be related to the operation and infection involves deep soft tissue (e.g., fascia and muscle) of the incision and at least one of the following:
  - Purulent drainage from the deep incision but not from the organ/space component of the surgical site
  - A deep incision spontaneously dehisces or is deliberately opened by a surgeon when the patient has at least one of the following signs or symptoms: fever (>38°C), localized pain, or tenderness, unless incision is culture negative
  - An abscess or other evidence of infection involving the deep incision is found on direct examination, during reoperation, or by histopathologic or radiologic examination
  - Diagnosis of deep incisional SSI made by a surgeon or attending physician
(IV) Organ space SSI: infection occurs within 30 days after the operation if no implant is left in place or within one year if implant is in place and the infection appears to be related to the operation and infection involves any part of the anatomy (e.g., organs) operation and at least one of the following:
  - Purulent drainage from a drain that is placed through a stab wound into the organ/space
  - Organisms isolated from an aseptically obtained culture of fluid or tissue in the organ/space
  - An abscess or other evidence of infection involving the organ/space that is found on direct examination, during reoperation, or by histopathologic or radiologic examination
  - Diagnosis of organ/space SSI made by a surgeon or attending physician

### 2.4. Study Variables and Data Collection Tools

Data were collected through face-to-face interviews and revision of hospital record charts. Proforma with structured questionnaire including the following variables was used for data collection.

- Age of the patient
- Age at marriage
- Education status
- Body mass index (during wound examination)
- Parity
- ANC visits
- Primary/previous cesarean
- Medical disorders in pregnancy (diabetes/hypertensive disorder/others)
- Emergency or elective cesarean
- Duration of surgery (from anesthesia to closure of the skin)
- Rupture of membrane (ARM/PROM) and its duration in hours
- Number of PV examinations
- Labor status
- Indication of cesarean section
- Blood loss during surgery in ml

### 2.5. Inclusion and Exclusion Criteria

#### 2.5.1. Inclusion Criteria (Case)

- Any surgical site infection following cesarean section in TUTH in study duration
- Surgical site infection defined according to CDC criteria
- Patients with SSI diagnosed on hospital stay or on follow-up after discharge

#### 2.5.2. Inclusion Criteria (Control)

- The immediate (before or after) cesarean section done in same day or a day before and after, in which the cesarean section of the case was done
- Women meeting above criteria and who do not develop SSI till 30^th^ POD (as per CDC)

#### 2.5.3. Exclusion Criteria (Case and Control)

- Women who did not response to follow-up or who did not show up for follow-up notification
- Patient who had undergone cesarean hysterectomy related to delivery
- Abdominal delivery after uterine rupture
- Women who had follow-up after 30 days
- Women following cesarean section in hospitals other than TUTH
- Women who do not want to take part in the study

### 2.6. Study Population and Framework

This is a hospital-based case-control type of study conducted for seven months from February 2019 to October 2019. The SSI cases among cesarean sections of 6 months from 27 February 2019 to 31 August 2019 participated in the study. There were total of 2446 deliveries in these six months out which 1135 (46.4%) had cesarean delivery (CD). All the women who developed surgical site infection as per CDC criteria and out of cesarean sections from February 2019 to August 2019 in department of obstetrics and gynecology in TUTH were included in the study as cases. Controls were selected as per inclusion criteria in 1 : 1 ratio.

All the women who had cesarean section in first 6 months of study duration were counselled regarding surgical site infections and its signs/symptoms. Counseling was done presurgery, during postpartum period in wards, and at time of discharge.

Surgical site infection was defined as per the CDC definition by CDC (1992). Those patients following cesarean section who developed SSI in ward or visited obstetric Outpatient Department (OPD) or emergency or got admitted in the ward for management were enrolled in the study as cases. Women who had undergone cesarean section on same day or one day before or after the cesarean section of SSI case and who did not develop SSI up to 30^th^ postoperative day were taken as control in the ratio 1 : 1. Among the possible controls, the first one who came to follow up was included in the study, and cases who did not follow up till 30^th^ postop day were excluded.

**Figure 1:**
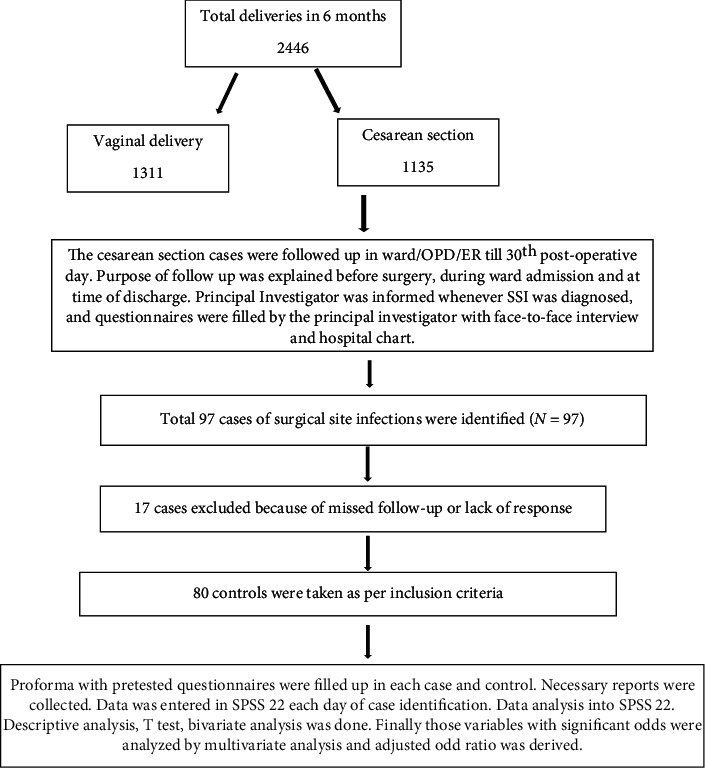
Study design/framework.

Consent was taken and detail history was recorded. Patients were examined, necessary investigations (as per hospital protocol) were sent, and proforma was filled. Both cases and controls were followed up till 30^th^ postoperative day and managed as per hospital protocol and all required information was collected. Detailed study design is shown in Figure 1.

### 2.7. Data Analysis

The data were properly coded, categorized, and checked for completeness, accuracy, clarity, and consistency by the principal investigator and supervisors before being entered into software for final analysis. Data was analyzed into SPSS 22. Predictor variables were recoded and dichotomized to perform analysis. Descriptive analysis was performed for each variable. Frequency and percentage were computed for categorical variable. Independent *T*-test was applied to compare the mean values of two groups. In bivariate analysis, binary logistic regression was employed to identify the one-to-one relationship between predictors and outcome variables. Before conducting multivariate analysis, multicollinearity was tested between the predictor's variables. All statistically significant variable levels of *p* < 0.05 in unadjusted analysis were included into the multivariate regression model. Finally, multivariate logistic regression was used to identify the relationship between host- and procedure-related risk factors with SSI after adjusting confounding variables. *p* value < 0.05 was considered as statistically significant.

### 2.8. Data Quality and Ethical Assurance

Data was collected by means of face-to-face interview as well as reviewing hospital record charts to get necessary information. To get informed consent and reliable data, a clear explanation of the purpose of the study was explained. Also, subjects were positive as their treatment was going together with the research.

The Tribhuvan University Teaching Hospital is an institution with ongoing multidisciplinary academic research. It has an institutional research board (IRB), which provided ethical clearance for this study before data collection after reviewing the proposal.

## 3. Results

**Figure 2:**
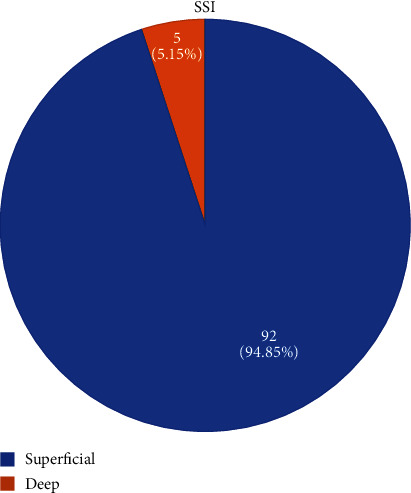
Types of SSI in the study.

There were 2446 deliveries in obstetric unit of TUTH in six months of study, out which 1135 (46.4%) had cesarean delivery (CD). Ninety-seven cases of surgical site infections were identified. The incidence rate of surgical site infection among cesarean section was calculated to be 8.54%. As shown in Figure 2, majority of SSI, i.e., 94.85% (*n* = 92), were superficial type and remaining 5.15% (*n* = 5) were deep without any deep incisional type of SSI. Among them, 80 cases and controls were taken as per inclusion criteria. Seventeen cases were not included as follow-ups were not continued by the patients. Sixty-nine percent of the SSI (*n* = 55) were diagnosed within 10^th^ postoperative days. Remaining 31% (*n* = 25) SSI were diagnosed after 10^th^ day. Maximum day of diagnosis was 26^th^ postoperative day.

Table 1 shows the host-related characteristics of patients with SSI following cesarean section compared with the control group. Majority of cases and controls were under the age of 26-29 years with mean age of case higher than controls, i.e., 26.88 ± 4.38 years and 24.93 ± 4.80 years, respectively, with significant *p* value of 0.008. Obesity and overweight were more prevalent in women with SSI than in the non-SSI group. In the SSI group, 17% (*n* = 14), 50% (*n* = 40), and 33% (*n* = 26) were found to have normal weight, overweight, and obesity, respectively, at the time of wound examination. Similarly, in controls, 51% (*n* = 41), 43% (*n* = 34), and 6% (*n* = 5) were found to have normal weight, overweight, and obesity, respectively, with statistically significant *p* value at <0.001.

Most of the women in both SSI group and non-SSI group were primipara. There were with 74% (*n* = 59) of primipara women in the SSI group and 60% (*n* = 48) in the non-SSI group with statistically not significant *p* value of 0.065.

Majority of women in both cases and controls had undergone primary CS. In the SSI group, 90% (*n* = 72) cases were primary CS and 10% (*n* = 8) were cases of repeat cesarean section. In the non-SSI group, 80% (*n* = 64) cases were primary CS, whereas 20% (*n* = 16) cases were repeat cesarean, i.e., previous CS cases. The result was not statistically significant at *p* value of 0.048.

There were more women with medical complications like diabetes (GDM/DM), HTN (pregnancy induced or chronic), and other disorders like hypothyroidism, anemia, fever, autoimmune disease like SLE, scleroderma in the SSI group, i.e., 53% (*n* = 42) out of total SSI cases. On contrary, 85% (*n* = 68) of women of the non-SSI group had no complication implying more the medical complications, more was the chance of developing SSI. The result was statistically significant at *p* value of <0.001 as depicted in the table below.

Table 2 shows procedure-related characteristics of patients with SSI following cesarean section compared with the control group. Emergency indication of cesarean section was more common in the SSI group with 95% occurrence (*n* = 76) compared to 81% (*n* = 56) in the non-SSI group, and elective cesarean section was more common in the non-SSI group, i.e., 19% (*n* = 15) as compared to the SSI group, i.e., 5% (*n* = 4). The result was statistically significant at *p* value of 0.013 as shown in Table 2.

Artificial rupture of membrane was done more frequently in the SSI group, i.e., 57% (*n* = 39) as compared to the non-SSI group, i.e., 48% (*n* = 27). However, the result was not statistically significant with *p* value of 0.310. The mean duration of rupture of membrane was longer in the SSI group, i.e., 9.5 ± 2.3 hours compared to 3.99 ± 1.9 hours in the non-SSI group implying longer the duration of membrane rupture more the chance of SSI.

Mean duration of membrane rupture was being more common in the SSI group than non-SSI, and duration was categorized as more than 18 hours and less than 18 hours, respectively. In the SSI group, there were a greater number of cases with membrane rupture duration longer than 18 hours, i.e., 13 (20%) as compared to the non-SSI group, i.e., 2 (4%), and it was statistically significant at *p* value of 0.005.

Total numbers of PV examinations, i.e., from admission to cesarean section, were categorized in two groups, i.e., up to 5 and more than 5. In the SSI group, there were more women who had PV examinations of more than 5 times, i.e., 54% (*n* = 43) as compared to the non-SSI group, i.e., 38% (*n* = 30). The result was significant at *p* value of 0.039.

Duration of surgery (i.e., from the time of anesthesia spinal/induction of anesthesia up to skin closure) was also found to be positively associated with occurrence of SSI. 62% (*n* = 50) of the SSI group and 45% (*n* = 36) of the non-SSI group had duration of surgery > 60 minutes at statistically significant *p* value of 0.026. And 38% (*n* = 30) of women in the SSI group and 55% (*n* = 44) of women in the non-SSI group had surgery duration of up to 60 minutes. Hence, the longer the surgery duration, the more the chance of SSI was expected in the study.

Most of the women were already in labor during cesarean section, i.e., 90% (*n* = 72) of the SSI group and 70% (*n* = 56) of the non-SSI group. Similarly, 10% (*n* = 8) of women in the SSI group and 30% (*n* = 24) in the non-SSI group had not gone into labor. This result was statistically significant *p* value of 0.002.

Total blood loss was up to 200 ml in 83% (*n* = 61) for SSI and 89% (*n* = 71) for the non-SSI group. It was found that the blood loss was in the range of 200-400 ml in 23% (*n* = 19) SSI as compared to 11% (*n* = 9) non-SSI group with statistically significant *p* value of <0.037.

Tables 3 and 4 show the bivariate and multivariate logistic regression model for host-, pregnancy-, and procedure-related risk factors among patients with SSI following cesarean section compared with the control groups.

After initial analysis, all statistically significant variable levels of *p* < 0.05 in unadjusted analysis were included into the multivariate regression model. Hence, in the multivariate logistic regression model, only few factors were seen to be positively associated with development of SSI in the host- and pregnancy-related risk factor.

In the multivariate regression model, women who were overweight with adjusted OR 4.11 (1.74-9.71) and *p* value of 0.001 and were in obese nutritional status with adjusted OR 15.72 (95% CI 4.60-53.67) and *p* value of <0.001 had higher chances of developing SSI than normal and underweight ones. Women having medical complication such as DM/HTN with adjusted 4.56 (1.60-12.99) and *p* value of 0.024 and other medical complications with adjusted OR 8.78 (2.68-28.80) and *p* value of <0.001 were significantly associated with SSI compared to those with no medical disorder.

In multivariate analysis, women who were already in labor had 6.52 times higher chance of developing SSI with adjusted OR 6.52 (1.17-36.38) at *p* value 0.032. Similarly, prolonged duration of membrane rupture, i.e., >18 hours, and a higher number of PV examination had higher chances of developing SSI with adjusted OR 8.38 (1.48-47.35) at *p* value 0.016 and 2.52 (1.01-6.30) at *p* value 0.046, respectively.

However, few of the significant variables in bivariate analysis like emergency CS, previous CS, prolonged duration of surgery, i.e., more than 60 minutes, labor dystocia cases, and those with more blood loss, i.e., more than 200 ml, did not show higher odds of SSI after multivariate analysis.

## 4. Discussion

Wound-related complication like surgical site infection following cesarean section is a major cause of morbidity and mortality, increasing both the duration of patient hospitalization and hospital costs [4]. It is the most common infection in surgical patients and constitutes 15% nosocomial infection [14]. With the rising trend in the cesarean deliveries worldwide, SSI is also increasing. Also, it is one of the frequently observed postoperative complications in the institute where the study was carried out. Most surgical site infections are caused by contamination of an incision with microorganisms present in patient's own body during surgery [15]. Infection caused by microorganisms from a source other than the patient's body following the surgery is less common [16]. Most surgical site infections are preventable [8]. Measures can be taken in the pre-, intra-, and postoperative phases of care to reduce the risk of infection [17, 18]. Proper postoperative surveillance of the cases with risk factors reduces the incidence and complications of wound infection [19].

### 4.1. Classification of SSI

CDC has classified surgical site infection into three categories, i.e., superficial incisional, deep incisional, and organ/space SSI. Different literatures mention superficial incisional type as the most common of all. In this study, out of 97 cases of SSI, 92, i.e., 94.8%, cases were superficial incisional and 5 cases, i.e., 5.2%, were deep incisional with no case of organ space infection.

### 4.2. Incidence of SSI

SSI rate after CS ranges from 3% to 15%, varying based on the population being studied, the methods used to monitor and identify the cases, and the use of appropriate antibiotic prophylaxis [1, 3, 20]. In this study, incidence rate of SSI was 8.54%. In study done in Nigeria, Jido et al. [6] reported the SSI rate of 9.1% following cesarean section. Previous study by Shrestha S et al. [5] in Nepal reported 12.6% incidence rate of SSI in 2014. Opøien et al. [21] in 2007 found that the total rate of SSI was 8.9%, with an observation period of 30 days postoperatively, compared to 1.8% registered at hospital discharge. In Patan Hospital, Pandit et al. [22] reported a rate of 2.76% as the incidence of wound infection among the cases of cesarean section from March 2002 to January 2003. This lower incidence rate might have been because of them considering only those SSI which developed during the hospital stay. Follow-ups of the cases were not conducted till 30^th^ POD as per CDC directives which might have resulted into lower rate.

### 4.3. Host-Related Risk factors

In the present study, *mean age* of SSI group was 26.88 ± 4.38 years compared to 24.81 ± 5.08 years. This means that women with SSI were relatively older than the non-SSI groups. With increasing age during pregnancy, risk of medical complications also increases [23].

*Obesity* has previously been reported to predict SSI via various possible factors, including the relative avascularity of adipose tissue [24]. Another factor may be technical difficulties of handling adipose tissue which can result into more trauma to the anterior abdominal wall, or difficulty in obliterating dead space in the fat-tissue of the abdominal wall [13]. This study also identified that women with obesity have higher risk of developing SSI than those with normal weight and underweight with adjusted OR 15.72 (4.60-53.67) at *p* value of <0.001.

*Antenatal checkupvisit* > 4 had higher odds, i.e., 1.94 (0.93-4.05), of developing SSI compared to those with less than or 4 ANC visits. However, result was not statistically significant with *p* value 0.074.

It was seen that *primipara* women were at higher risk of developing SSI with OR 1.87 (0.96-3.66) as compared to multipara women; however, the result was not statistically significant with *p* value 0.066.

It is assumed that women with *previous CS* have increased risk of surgical site infection due to poor healing of previous scarred tissue in which incision is repeated, relative avascularity, more blood loss during surgery, and longer surgery duration [23]. But in this study, previous CS had 0.38 (0.14-0.99) odds of having SSI as compared to primary CS. Even though the result was not statistically significant at *p* value of 0.337 on multivariate analysis, previous CS seems to have lower odds of developing SSI in comparison to primary CS. Also in a study of SSI following cesarean, by Jido and Garba [6], forty-one (93.1%) of the cases were primary CSs compared to 327 (74.1%) of the controls, i.e., SSI was more frequent in primary CS than previous CS.

In this study, women having *medical complication* such as DM/HTN with AOR 4.75(1.69-13.32) and *p* value of 0.003 and other medical complications with AOR 9.38 (95% CI 2.89-30.46) and *p* value of 0.001 were significantly associated with SSI compared to those having no medical disorder. *Other medical complications* included hypothyroidism, anemia, heart disease, respiratory tract infections with fever, and immune-mediated disorder like scleroderma. There were 8 cases of hypothyroidism, 6 cases of anemia, 3 cases of respiratory tract infections with fever, 2 cases of heart disease, and 1 case of scleroderma.

### 4.4. Procedure-Related Risk factors

*Emergency CS* had 4.38 times higher odds of SSI than elective surgery, i.e., OR 4.38 (CI 1.38-13.86, *p* value 0.012). However, on multivariate analysis, the adjusted odds ratio was omitted due to multicollinearity effect of multiple variables.

The mean duration of *rupture of membrane* in cases was 9.5 ± 19.11 hours, whereas for controls, it was 3.98 ± 13.54 hours implying that mean duration was higher for cases. Hence, duration was categorized as 18 hours or less and more than 18 hours. The duration of rupture for more than 18 hours was predictive of SSI on both bivariate and multivariate analysis with AOR 8.38 (1.48-47.35) at *p* value 0.016. However, there was no significant difference between spontaneous and artificial rupture of the membrane among the cases and control with *p* value of 0.311. Also, no study has compared between artificial and spontaneous rupture. In cesarean section, nonsterile amniotic fluid may act as a transport medium by which bacteria get to the uterine and skin incisions leading to chorioamnionitis and its sequelae like SSI.

This study showed that subject with *more than five PV examination before the procedure* had higher risk of developing SSI with AOR 2.52 (1.01-6.30), *p* value of 0.046 compared to that of less than 5 times. With increased number of PV examination, there are chances of more contamination from vagina to endometrium and hence uterine wall, which will ultimately traverse to incision site during cesarean delivery. Similarly, Saeed and et al. [25] concluded that there was increased risk of SSI for women who had ≥5 vaginal examinations (AOR, 3.24; 95% CI, 0.92-11.41). In a study by Mpogoro et al. [7], they concluded that multiple vaginal examinations (HR: 2.5; 95% CI, 1.2-5.1; *p* = 0.011) was one of the causes for SSI.

*Surgery duration for more than 60 minutes* was associated with higher risk of surgical site infection with adjusted OR of 2.12 (95% CI 0.91-4.90); however, the result was not statistically significant on multivariate analysis with *p* value 0.080.

Compared to those who were not in labor, *women who were already in labor* during cesarean section (including all stages of labor) had higher odds of developing SSI, i.e., AOR 6.52 (1.17-36.38), at significant *p* value 0.032 on multivariate analysis. *Labor status* increases the chance of multiple PV examinations, rupture of membrane, prolonged rupture, prolonged latent phase, and other labor dystocia, which indirectly increases the chance of SSI.

*Meconium-stained liquor* was the most common indication in both the SSI and non-SSI groups. Hence, cases were divided in terms of indication as meconium stained and nonmeconium stained. Indication as meconium was more common in the SSI group as compared to non-SSI; however, the result was not statistically significant with *p* value of 0.072. On bivariate analysis, meconium stained had higher odds of developing SSI with OR 1.92 (0.93-3.95) compared to nonmeconium-stained indications.

Also in this study, *labor dystocia* including prolonged latent phase of labor, nondescent of head, and nonprogress of labor was another common indication of CS in the SSI group. On multivariate analysis, there was higher odds of developing SSI among labor dystocia group, i.e., AOR 1.45 (0.45-4.62); however, the result was not statistically significant with *p* value 0.531.

*Blood loss* had higher odds of developing SSI, i.e., 2.45 (1.03-5.82), with significant *p* value of 0.013, but the result was not significant on multivariate analysis.

## 5. Conclusion

Surgical site infection following cesarean section is a common complication with incidence of 8.54% in TUTH,IOM, Nepal. Multiple risk factors like increasing age, obesity, medical complications during pregnancy, initiation of labor during cesarean section, prolonged duration of rupture of membrane for more than 18 hours, and more than five PV examination increase the chance of surgical site infection after cesarean section. Hence, obstetrician should consider earlier or more frequent postoperative follow-up in patients with these risk factors. Obstetrician should try to avoid preventable risk factors to reduce incidence of surgical site infection following cesarean section.

## Figures and Tables

**Table 1:** Host-related characteristics of patients with SSI following cesarean section compared with control group.

Respondent's characteristics	Case (SSI) *n* (%)	Control (non-SSI) *n* (%)	*p* value
Age			0.128
15-20	5 (6)	11 (14)	
21-25	22 (28)	29 (36)	
26-30	40 (50)	33 (41)	
>30	13 (16)	7 (9)	
Education level			0.664
Lower primary	30 (38)	25 (31)	
Secondary	21 (26)	25 (31)	
Graduate or above	29 (36)	30 (38)	
BMI			<0.001^∗^
Normal	14 (17)	41 (51)	
Overweight	40 (50)	34 (43)	
Obesity	26 (33)	5 (6)	
Parity			0.065
Primipara	59 (74)	48 (60)	
Multipara	21 (26)	32 (40)	
Site of ANC visits			1.00
TUTH	67 (84)	67 (84)	
Outside TUTH	13 (16)	13 (16)	
ANC visits			0.074
≤4 times	15 (19)	25 (32)	
>4 times	63 (81)	54 (68)	
Previous vs primary CS			0.048
Previous CS	7 (9)	16 (20)	
Primary CS	73 (91)	64 (80)	
Complication during ANC			<0.001^∗^
None	38 (47)	68 (85)	
DM/HTN	22 (28)	7 (9)	
Others	20 (25)	5 (6)	

^∗^Denotes statistically significant at *p* < 0.05.

**Table 2:** Procedure-related characteristics of patients with SSI following cesarean section compared with control group.

Procedure-related characteristics	Case (SSI) *n* (%)	Control (non-SSI) *n* (%)	*p* value^1^
Type of CS			0.013^∗^
Emergency	76 (95)	65 (81)	
Elective	4 (5)	15 (19)	
Membrane rupture			0.310
Artificial	39 (57)	27 (48)	
Spontaneous	29 (43)	29 (52)	
Duration of membrane rupture			
≤18 hours	50 (80)	54 (96)	0.005
18 hours	13 (20)	2 (4)	
Number of PV examination			
≤5 times	37 (46)	50 (62)	0.039^∗^
>5 times	43 (54)	30 (38)	
Duration of surgery			0.026^∗^
≤60 minutes	30 (38)	44 (55)	
>60 minutes	50 (62)	36 (45)	
Status of labor			0.002^∗^
NIL	8 (10)	24 (30)	
ESOL/ASOL/SSOL	72 (90)	56 (70)	
Blood loss during surgery (ml)			<0.037^∗^
100-200	61 (83)	71 (89)	
250-400	19 (23)	9 (11)	

^1^Chi-square test and Fisher exact test. ^∗^Denotes statistically significant at *p* < 0.05.

**Table 3:** Bivariate and multivariate logistic regression model for host- and pregnancy-related risk factor.

Host- and pregnancy-related characteristics	Bivariate analysis		Multivariate analysis	
	Unadjusted OR (95% CI)	*p* value	Adjusted OR (95% CI)	*p* value
*Age*				
≤30	Ref			
>30	2.02 (0.76-5.37)	0.157		
*Education level*				
Up to lower primary	1.24 (0.59-2.59)	0.565		
Secondary	0.86 (0.40-1.88)	0.722		
Graduate or above	Ref			
*BMI*				
Normal	Ref			
Overweight	3.44 (1.61-7.36)	0.001^∗^	4.11 (1.74-9.71)	0.001^∗^
Obesity	15.22 (4.90-47.29)	<0.001^∗^	15.72 (4.60-53.67)	<0.001
*Parity*				
Primi	Ref			
Multi	0.53 (0.27-1.04)	0.066		
*ANC visits*				
≤4 times	Ref			
>4 times	1.94 (0.93-4.05)	0.074		
*Previous CS*				
Yes	0.38 (0.14-0.99)	0.048^∗^	0.599 (0.21-1.70)	0.337
No	Ref			
*Medical complication*				
None	Ref		Ref	
DM/HTN	5.62 (2.19-14.37)	<0.001^∗^	4.75 (1.69-13.32)	0.003^∗^
Others	7.15 (2.48-20.60)	<0.001^∗^	9.38 (2.89-30.46)	0.001^∗^

^∗^Denotes statistically significant at *p* < 0.05.

**Table 4:** Bivariate and multivariate logistic regression model for procedure-related risk factor.

Procedure-related characteristics	Bivariate analysis		Multivariate analysis	
	Unadjusted OR (95% CI)	*p* value	Adjusted OR (95% CI)	*p* value
*Type of CS*				
Emergency	4.38 (1.38-13.86)	0.012^∗^		
Elective	Ref			
*Membrane rupture*				
Artificial	1.44 (0.70-2.94)	0.311		
Spontaneous	Ref			
*Duration of rupture*				
≤18 hours	Ref		Ref	0.016∗
>18 hours	7.02 (1.50-32.66)	0.013^∗^	8.38 (1.48-47.35)	
*Number of PV examination*				
≤5 times	Ref		Ref	
>5 times	1.93 (1.03-3.64)	0.040^∗^	2.52 (1.01-6.30)	0.046^∗^
*Duration of CS*				
≤60 minutes	Ref		Ref	
>60 minutes	2.03 (1.08-3.83)	0.027^∗^	2.12 (0.91-4.90)	0.080
*Stage of labor*				
NIL	Ref		Ref	
ESOL/ASOL/SSOL	3.85 (1.61-9.23)	0.002^∗^	6.52 (1.17-36.38)	0.032^∗^
*Meconium*				
Yes	1.92 (0.93-3.95)	0.075		
No	Ref			
*Labor dystocia*				
Yes	1.39 (1.12-1.78)	0.013^∗^	1.45 (0.45-4.62)	0.531
No	Ref		Ref	
*Blood loss (ml)*				
100-200	Ref		Ref	
250-400	2.45 (1.03-5.82)	0.041^∗^	2.32 (0.71-7.45)	0.159

^∗^Denotes statistically significant at *p* < 0.05.

## Data Availability

Data is available upon request from the corresponding author.

## Acronyms

ANC: Antenatal care
AOR: Adjusted odds ratio
ARM: Artificial rupture of membrane
ASOL: Active stage of labor
CD: Cesarean delivery
CDC: Centers for Disease Control and Prevention
CS: Cesarean section
DM: Diabetes mellitus
GDM: Gestational diabetes mellitus
HAI: Healthcare-associated infection
HTN: Hypertension
IOM: Institute of Medicine
LMI: Low- and middle-income countries
LPOL: Latent phase of labor
NDOH: Nondescent of head
NIL: Not in labor
NPOL: Nonprogress of labor
OR: Odds ratio
PIH: Pregnancy-induced hypertension
PROM: Premature rupture of membrane
PV: Per vaginal
SROM: Spontaneous rupture of membrane
SSI: Surgical site infection
SSOL: Second stage of labor
TUTH: Tribhuvan University Teaching Hospital
WHO: World Health Organization.

## References

[1] Zuarez-Easton S., Zafran N., Garmi G., Salim R. (2017). Postcesarean wound infection: prevalence, impact, prevention, and management challenges. *International Journal of Women's Health*.

[2] Declercq E., Barger M., Cabral H. J. (2007). Maternal outcomes associated with planned primary cesarean births compared with planned vaginal births. *Obstetrics & Gynecology*.

[3] Horan T. C., Gaynes R. P., Martone W. J., Jarvis W. R., Grace E. T. (1992). CDC definitions of nosocomial surgical site infections, 1992: a modification of CDC definitions of surgical wound infections. *American Journal of Infection Control*.

[4] Protocol for surgical site infection surveillance with a focus on settings with limited resources [Internet]. https://www.who.int/publications/i/item/protocol-for-surgical-site-infection-surveillance-with-a-focus-on-settings-with-limited-resources.

[5] Shrestha S., Shrestha R., Shrestha B., Dongol A. (2014). Incidence and risk factors of surgical site infection following cesarean section at Dhulikhel Hospital. *Kathmandu University Medical Journal*.

[6] Jido T., Garba I. (2012). Surgical-site infection following cesarean section in Kano, Nigeria. *Annals of Medical and Health Sciences Research*.

[7] Mpogoro F. J., Mshana S. E., Mirambo M. M., Kidenya B. R., Gumodoka B., Imirzalioglu C. (2014). Incidence and predictors of surgical site infections following caesarean sections at Bugando Medical Centre, Mwanza, Tanzania. *Antimicrobial Resistance and Infection Control*.

[8] Lakhan P., Doherty J., Jones M., Clements A. (2010). A systematic review of maternal intrinsic risk factors associated with surgical site infection following caesarean sections. *Healthcare Infection*.

[9] Farret T. C. F., Dallé J., da Silva M. V., Riche C. V. W., Antonello V. S. (2015). Risk factors for surgical site infection following cesarean section in a Brazilian Women’s Hospital: a case-control study. *Brazilian Journal of Infectious Diseases*.

[10] Killian C. A., Graffunder E. M., Vinciguerra T. J., Venezia R. A. (2001). Risk factors for surgical-site infections following cesarean section. *Infection Control & Hospital Epidemiology*.

[11] Gong S. P., Guo H. X., Zhou H. Z., Chen L., Yu Y. H. (2012). Morbidity and risk factors for surgical site infection following cesarean section in Guangdong Province, China. *Journal of Obstetrics and Gynaecology Research*.

[12] Shree R., Park S. Y., Beigi R. H., Dunn S. L., Krans E. E. (2016). Surgical site infection following cesarean delivery: patient, provider, and procedure-specific risk factors. *American Journal of Perinatology*.

[13] Dagshinjav N., Tudevdorj E., Davaasuren M., Gurjav N. (2017). Risk factors for sepsis following cesarean section in Ulaanbaatar: a case-control study. *Central Asian Journal of Medical Sciences*.

[14] McKibben L., Horan T., Tokars J. I. (2005). Guidance on public reporting of healthcare-associated infections: recommendations of the Healthcare Infection Control Practices Advisory Committee. *American Journal of Infection Control*.

[15] Kaplan N. M., Smadi A. A., Al-Taani M. I., El-Qudah M. A. (2003). Microbiology of wound infection after caesarean section in a Jordanian hospital. *Eastern Mediterranean Health Journal*.

[16] Wloch C., Wilson J., Lamagni T., Harrington P., Charlett A., Sheridan E. (2012). Risk factors for surgical site infection following caesarean section in England: results from a multicentre cohort study. *BJOG: An International Journal of Obstetrics & Gynaecology*.

[17] Jenks P. J., Laurent M., McQuarry S., Watkins R. (2014). Clinical and economic burden of surgical site infection (SSI) and predicted financial consequences of elimination of SSI from an English hospital. *Journal of Hospital Infection*.

[18] Stevens D. L., Bisno A. L., Chambers H. F. (2005). Practice guidelines for the diagnosis and management of skin and soft-tissue infections. *Clinical Infectious Diseases*.

[19] Bärwolff S., Sohr D., Geffers C. (2006). Reduction of surgical site infections after Caesarean delivery using surveillance. *Journal of Hospital Infection*.

[20] Schneid-Kofman N., Sheiner E., Levy A., Holcberg G. (2005). Risk factors for wound infection following cesarean deliveries. *International Journal of Gynecology & Obstetrics*.

[21] Opøien H. K., Valbø A., Grinde-Andersen A., Walberg M. (2007). Post-cesarean surgical site infections according to CDC standards: rates and risk factors. A prospective cohort study. *Acta Obstetricia et Gynecologica Scandinavica*.

[22] Pandit A., Sharma P., Yangzom K. (2013). Incidence of caesarean wound infection in Patan Hospital, Nepal. *Journal of Nepal Medical Association*.

[23] Miller E. S., Hahn K., Grobman W. A. (2013). Consequences of a primary elective cesarean delivery across the reproductive life. *Obstetrics & Gynecology*.

[24] St V., Lamoutte C., De S., Verdeja A. (2000). Wound infection after cesarean: effect of subcutaneous tissue thickness. *Obstetrics and Gynecology*.

[25] Saeed K. B., Corcoran P., O’Riordan M., Greene R. A. (2019). Risk factors for surgical site infection after cesarean delivery: a case-control study. *American Journal of Infection Control*.

